# CD8^+^ Tissue‐Resident Memory T Cells in Health and Disease

**DOI:** 10.1002/mco2.70838

**Published:** 2026-07-05

**Authors:** Yu‐Han Ai, Ting‐Ting Xu, Sen Lin, Meng‐Yu Xiao, Yun‐Xia Lin, Hao‐Wen Ma, Yue‐Yang Liu, Yao‐Jie Wang, Lu‐Ning Yin, Mei Huang, Yong‐Da Liu, Chao Zhu, Xiao‐Ting Zhu, Ji‐Hang Yuan

**Affiliations:** ^1^ Department of Medical Genetics Naval Medical University Shanghai China; ^2^ Center for Disease Control and Prevention of Shanxian County Heze China; ^3^ Department of Rheumatology and Immunology The Third Affiliated Hospital of Naval Medical University Shanghai China; ^4^ Department of Anatomy and Physiology Shanghai Jiao Tong University School of Medicine Shanghai China

**Keywords:** antitumor immunity, autoimmune disease, tissue immunity, tissue‐resident memory T cells, viral infection

## Abstract

CD8^+^ tissue‐resident memory T (T_RM_) cells play a pivotal role in local immune regulation. These noncirculating T cells are uniquely positioned to orchestrate rapid site‐specific immune responses, making them central to understanding tissue‐specific immunity. Prior work has demonstrated that CD8^+^ T_RM_ cells possess dual functions: they are essential for immune defense against pathogens and malignancies, yet also drive pathological inflammation in autoimmune disorders. Furthermore, emerging evidence has begun to elucidate the intrinsic molecular mechanisms controlling this functional duality and to develop intervention strategies for its precise modulation. Here, we review current knowledge of the origin, transcriptional regulation, phenotypic characteristics, and functions of CD8^+^ T_RM_ cells. We discuss how T_RM_ cells correlate with clinical outcomes in diverse cancer types. Furthermore, we explore immune checkpoint inhibitors and other immunotherapies that target T_RM_ cells and their therapeutic implications from preclinical and translational human research. This review provides a comprehensive understanding of the molecular and cellular mechanisms governing T_RM_ cell activity and details their roles in health and disease. Ultimately, the framework of tissue immune networks reveals that leveraging T_RM_ cell development, tissue residency, and metabolism processes enables the design of innovative treatments for these diseases.

## Graphical abstract

CD8^+^ T_RM_ cells are at the center of immunity, mediating potent local innate and adaptive immune responses. While T_RM_ cells mediate protective immunity against infections and tumors, they also contribute to pathological inflammation in autoimmune conditions. CD8^+^ T_RM_ cells strongly correlate with clinical outcomes, including prolonged patient survival and reduced cancer recurrence. As key mediators of durable immune protection, CD8^+^ T_RM_ cells are emerging as critical targets for next‐generation immunotherapies, offering potential strategies to enhance T cell persistence, disease control, and long‐term therapeutic efficacy. Targeting CD8^+^ T_RM_ could unlock new avenues for more effective and sustained immunotherapy.

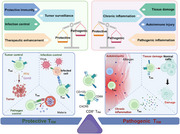

## 1 Introduction

The identification and characterization of tissue‐resident memory T (T_RM_) cells represent a fascinating milestone in immunology research. The journey began in 1999 when Federica Sallusto classified circulating memory CD8^+^ T cells into two subsets: central memory T (T_CM_) cells and effector memory T (T_EM_) cells [1, 2]. A pivotal advance came in 2001, when David Masopust and colleagues demonstrated that pathogen‐specific T cells could migrate to diverse tissues (e.g., bone marrow, lung, intestine, and kidney) and persist for months as functional effector memory cells [3]. Subsequent studies revealed that a subset of these tissue‐localized T cells did not recirculate, uncovering the novel tissue‐resident properties of memory T cells [4, 5]. In 2009, Thomas Gebhardt's team formally introduced the term “tissue‐resident memory T cells”, a nomenclature now universally adopted [6]. Over the past decade, extensive research has established that T_RM_ cells are widely distributed across multiple tissues and organs in both humans and mice [7, 8, 9, 10], underscoring their biological and clinical significance.

CD8^+^ memory T cells are now broadly categorized into three populations: T_CM_, T_EM_, and T_RM_ cells. T_CM_ cells, which express lymph node homing receptors CCR7 and CD62L, are highly proliferative and long‐lived, serving as a reservoir capable of robust expansion. Conversely, T_EM_ cells express low levels of CCR7 and CD62L, traffic through spleen, blood, and peripheral tissues, and maintain immediate effector potential [1, 3]. Notably, T_RM_ cells are characterized by the downregulation of CCR7 and CD62L, which prevents tissue egress, and the upregulation of adhesion molecules, including CD69, CD103, CD49a, CXCR3, and CXCR6, which collectively promote tissue retention [11, 12].

T_RM_ cells reside in a variety of tissues, including the skin, brain, lung, liver, kidney, salivary glands, stomach, pancreas, gut, and reproductive tract [6, 13, 14, 15, 16, 17, 18, 19]. The establishment of this tissue‐resident program across various organs is governed by a unique gene expression program driven by transcription factors such as Runx3, Blimp1, Hobit, Bhlhe40, and T‐box transcription factors [20, 21, 22, 23]. CD8^+^ T_RM_ cells occupy a central position in the immune response, coordinating potent innate and adaptive immune reactions locally [24]. Beyond direct cytotoxicity, CD8^+^ T_RM_ cells engage in crosstalk with other immune cells, amplifying local immune surveillance [25]. CD8^+^ T_RM_ cells are widely recognized for mediating beneficial antiviral and antitumor immune responses. But when CD8^+^ T_RM_ cells become dysregulated, they can cause inflammation and tissue damage, which is frequently observed in autoimmune diseases [26].

Clinically, the density of tumor‐infiltrating T_RM_ cells correlates strongly with disease stage and patient prognosis, underscoring their significance in antitumor immunity [27]. In human cancers, CD103^+^ CD8^+^ T_RM_ cells have been linked to prolonged survival across multiple malignancies, including breast, colorectal, endometrial, ovarian, cervical, and bladder cancers [28, 29, 30, 31, 32, 33]. Consequently, CD8^+^ T_RM_ cells have emerged as promising targets for immunotherapy. For instance, effective vaccines against the hepatitis B virus (HBV) and malaria aim to elicit robust activation of CD8^+^ T_RM_ cells [34, 35]. On the other hand, therapeutic strategies that disrupt residency signals or block pro‐inflammatory cytokines from CD8^+^ T_RM_ cells can alleviate autoimmune disease progression [36, 37, 38]. Therefore, a deeper understanding of CD8^+^ T_RM_ cells will pave the way for novel therapeutic interventions.

Here, we discuss the mechanisms underlying CD8^+^ T_RM_ cells development, homing, localization, and differentiation, and highlight their protective or pathogenic roles in various diseases. We summarize their physiological functions and phenotypic heterogeneity across tissues and disease contexts. Finally, we discuss strategies to modulate T_RM_ cell formation and function, aiming to amplify their antipathogen and antitumor efficacy or to mitigate their pathogenic and pro‐inflammatory effects, with the ultimate goal of improving clinical outcomes.

## 2 CD8^+^ T_RM_ Cells: The Local Sentinels of Tissue Immunity

CD8^+^ T_RM_ cells stand as the critical local sentinels of tissue immunity, establishing long‐term residency within peripheral tissues to mediate rapid and robust frontline defense against invading pathogens and emerging malignancies. Here, we systematically summarize their core phenotypic characterization, the processes governing their priming and fate determination, and the key molecular and cellular mechanisms underlying their differentiation.

### 2.1 Characterization of Tissue‐Resident Memory T Cells

**FIGURE 1:**
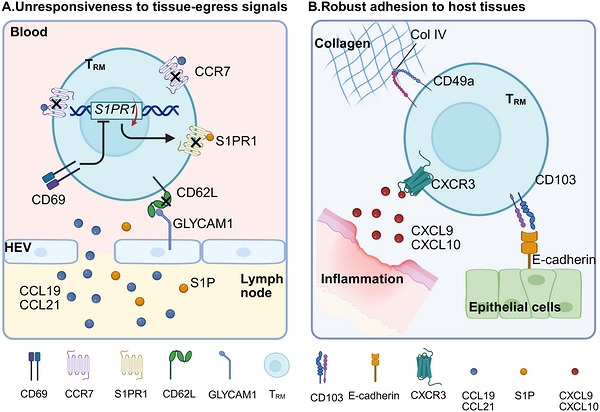
The residence mechanism of T_RM_ cells. (A) Unresponsiveness to tissue‐egress signals: T_RM_ cells downregulate their responsiveness to S1P gradient by CD69‐mediated degradation of S1PR1, thereby inhibiting tissue egress. Additionally, they exhibit low expression of lymph node homing receptors CCR7 and CD62L (L‐selectin), preventing their re‐entry into secondary lymphoid organs. (B) Robust adhesion to host tissues: T_RM_ cells maintain tissue residency through integrin α1β1 (CD49a)‐mediated recognition of basement membrane collagen IV networks, while their CXCR3 expression enables targeted migration to inflammatory sites in response to chemokines CXCL9/10.

Cytotoxic CD8^+^ T cells play a pivotal role in controlling invading pathogens and suppressing tumor outgrowth. CD8^+^ T_RM_ cells sustain long‐term persistence and robust effector function within diverse tissue microenvironments across health and disease settings, via a repertoire of specialized molecular adaptations. A characteristic feature of these cells is the selective upregulation of core surface markers that collectively mediate stable tissue retention and pathogen‐resistant effector responses. Critical retention markers include CD103 (αE integrin), which binds E‐cadherin on epithelial cells, CD69, which regulates tissue egress pathways, and CD49a (α1 integrin/VLA‐1), which mediates stromal interactions [11, 39, 40, 41, 42, 43, 44, 45, 46]. The retention of T_RM_ cells in tissues relies on two major mechanisms: evading signals that would otherwise drive them into blood or lymphoid tissues, and adhering to their local tissue environment (Figure 1). Notably, T_RM_ cells exhibit low expression of sphingosine‐1‐phosphate (S1P) receptors (e.g., S1PR1), which typically mediate T cell chemotaxis [47]. CD69, a type II transmembrane C‐type lectin, reinforces this insensitivity by binding the TM4 domain of S1PR1, triggering its endocytosis and degradation [44, 45, 47, 48, 49]. This disruption of S1P gradient sensing effectively blocks lymphocyte egress. Furthermore, T_RM_ cells lack CD62L (L‐selectin) and CCR7, receptors essential for tissue exit and lymph node homing [50, 51]. CCR7 and its ligands (CCL19/CCL21) normally guide T cells and dendritic cells (DCs) to lymphoid organs—a process from which T_RM_ cells are excluded [2, 52, 53].

The precise localization of T_RM_ cells is determined by tissue‐specific retention molecules. For instance, the most extensively characterized T_RM_ populations reside in epithelial tissues and express CD103, which mediates adhesion through binding to E‐cadherin on epithelial cells [54, 55]. Similarly, CD49a enhances tissue retention and survival by engaging extracellular matrix components such as collagen and laminin [40, 56, 57]. Furthermore, CD8^+^ T_RM_ cells upregulate CXCR3, a chemokine receptor that directs their migration toward inflammatory sites enriched with ligands like CXCL9 and CXCL10 [11, 58]. This CXCR3‐chemokine axis plays a pivotal role in recruiting T_RM_ cells to the tissue microenvironment, underscoring its functional importance in antitumor immunity.

### 2.2 CD8^+^ T Cell Priming and the Determination of T_RM_ Fate

CD8^+^ T cells are categorized into naive, effector, and memory subsets based on their differentiation status. Naive T cells (T_N_) remain in a resting state until antigen encounter. Following antigen stimulation, they differentiate into effector T cells (T_EFF_) [59, 60], a subset of which subsequently transition into quiescent memory T cells to mediate long‐term immune protection [59, 61, 62, 63]. Within the circulating T_EFF_ cells compartment, two distinct cell states are recognized: terminal effector cells—short‐lived, highly cytotoxic cells characterized by elevated expression of KLRG1, T‐bet, and Blimp1; memory precursor cells—progenitors of stable memory populations, marked by high expression of IL‐7Rα, ID3, and TCF1 [64]. A parallel phenotypic and functional dichotomy exists among tissue‐infiltrating T_EFF_ cells in nonlymphoid tissues (NLTs). CD8^+^ circulating memory T cells (T_CIRCM_) are critical for durable tumor control, as they can rapidly proliferate upon antigen re‐exposure to generate large effector cell populations. The T_CIRCM_ pool itself is heterogeneous, comprising subsets with distinct epigenetic, transcriptional, and translational profiles [65]. However, under persistent antigen exposure in tumors, even tumor‐reactive T cells often progress to a terminally dysfunctional or exhausted state, compromising their antitumor efficacy [60, 66].

T_RM_ cells are believed to originate from primary memory precursors, including naive T cells and early effector T cells, or from circulating memory precursor cells [64, 67]. Previous studies have demonstrated that inflammatory signals within tissue microenvironments can drive the differentiation of these memory precursors into mature CD8^+^ T_RM_ cells. For many years, the prevailing model of T_RM_ cell development followed the “local divergence” hypothesis, which proposed that lineage commitment occurred exclusively within tissues. However, emerging evidence now supports a competing “systemic divergence” model. This alternative paradigm suggests that circulating T cells can undergo preconditioning in lymphoid organs, acquiring a T_RM_ cell‐biased differentiation program before tissue entry. This model suggests the existence of a distinct population of T_RM_ cell‐poised T cells within the lymphoid compartment [18, 65, 68, 69, 70, 71, 72].

### 2.3 Unraveling the Path to CD8^+^ T_RM_ Differentiation

**FIGURE 2:**
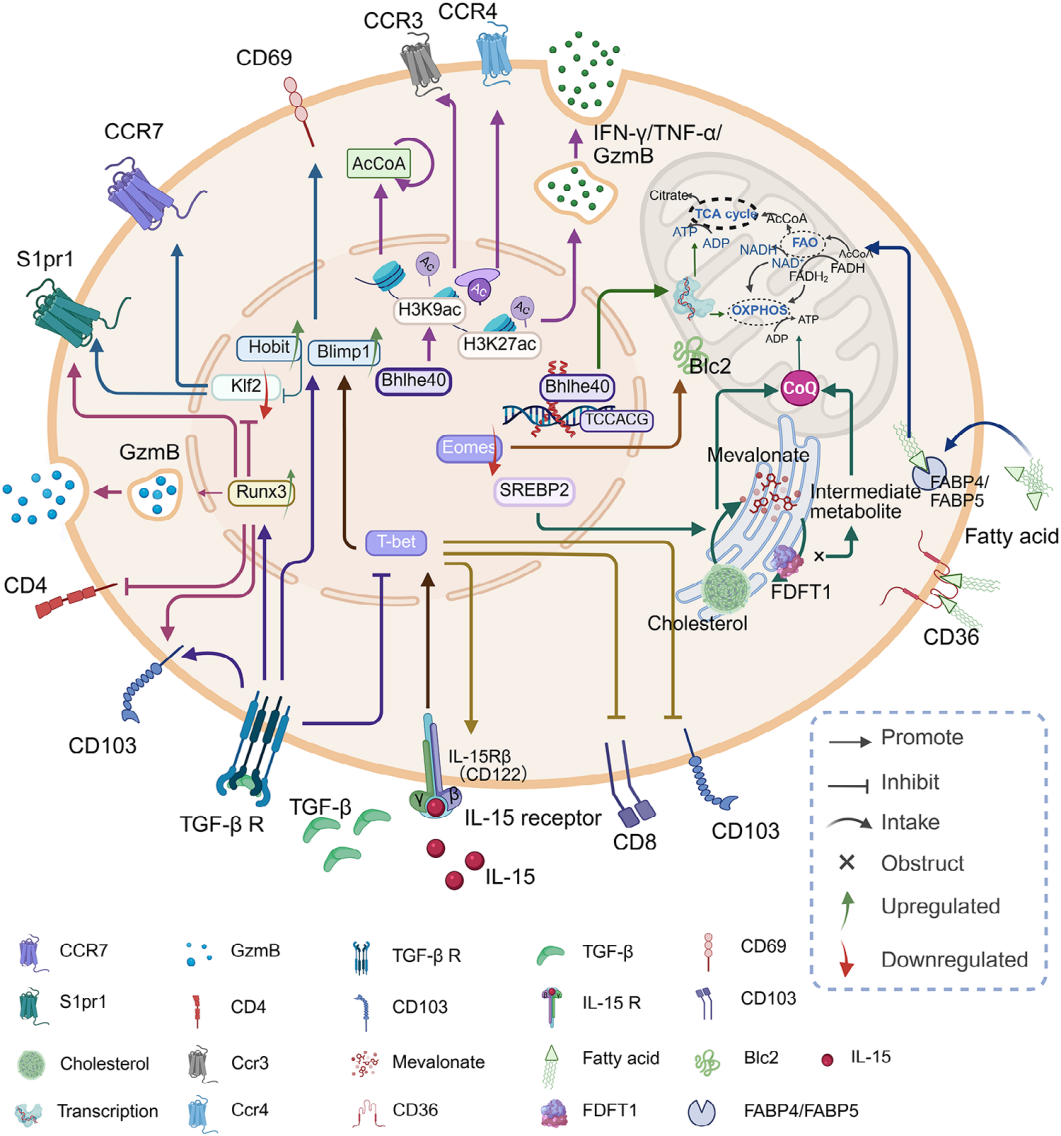
Differentiation of CD8^+^ T_RM_ cells. The differentiation of CD8^+^ T_RM_ cells is coordinately regulated by multiple transcription factors, cytokines, and metabolic pathways. The core transcription factor Hobit/Blimp1 promotes T_RM_ cell formation by suppressing the KLF2‐S1pr1‐CCR7 pathway. Runx3 enhances the expression of core residency genes, such as CD103, through TGF‐β‐dependent transcriptional mechanisms. Bhlhe40 regulates the expression of effector molecules by maintaining histone acetylation levels. Although the expression levels of T‐bet/Eomes are reduced in T_RM_ cells, they remain critical for the long‐term survival of these cells. TGF‐β signaling upregulates the expression of CD103 and Runx3 while inducing the low expression of T‐bet/Eomes. IL‐15 signaling upregulates Hobit expression in a T‐bet‐dependent manner, thereby promoting T_RM_ cell differentiation. In terms of metabolic pathways, the SREBP2‐CoQ axis supports mitochondrial respiration, and FABP4/5 mediates the uptake of exogenous fatty acids to sustain the long‐term survival of T_RM_ cells. These mechanisms collectively regulate the tissue residency and functional maintenance of CD8^+^ T cells.

The differentiation of CD8^+^ T_RM_ cells follows a tightly controlled trajectory regulated by transcriptional networks, cytokine signaling, and metabolic reprogramming (Figure 2). These core programs guide naïve T cells to adopt the tissue‐resident memory lineage while also directing their tissue tropism and maturation into functional CD8^+^ T_RM_ cells.

#### 2.3.1 Transcriptional Regulation

Hobit and Blimp1 form a core transcriptional program governing tissue residency in CD8^+^ T cells [22]. These factors are essential for T_RM_ cell differentiation. They upregulate CD69 and repress KLF2, which in turn suppresses KLF2 targets S1PR1 and CCR7—key mediators of tissue egress [22, 73]. Simultaneously, they divert precursor cells from a circulating memory fate toward the T_RM_ lineage. Critically, Hobit and Blimp1 act cooperatively to silence genes involved in tissue exit [74], consolidating their role as master regulators of T_RM_ cell retention.

Extensive research has established Runt‐related transcription factor 3 (Runx3) as a critical transcriptional regulator of CD8^+^ T cell biology. During thymic development, Runx3 directs CD8^+^ T cell lineage specification by simultaneously activating CD103 expression and repressing CD4 transcription [75]. In peripheral CD8^+^ T cells, Runx3 is indispensable for the acquisition of cytotoxic effector functions [76, 77]. The epigenetic regulation of Runx3 through DNA methylation further modulates CD8^+^ T cell differentiation and tumor infiltration capacity [78]. Mechanistically, Runx3 promotes T_RM_ cell formation through a transforming growth factor‐β (TGF‐β)‐dependent pathway that enhances chromatin accessibility at T_RM_‐specific genetic loci [79]. This process strictly requires intact TGF‐β signaling in vivo [80]. Overall, overexpression of Runx3 enhanced CD8^+^ T_RM_ cells abundance, expression of certain core tissue‐resident genes (e.g., CD69 and CD103), while further inhibiting the expression of core circulation genes (e.g., KLF2, CCR7, and S1PR1) [21]. These findings have direct clinical relevance, as Runx3 is essential for maintaining tumor‐resident CD8^+^ T_RM_ cell populations, highlighting its potential as a therapeutic target for modulating immune responses in diverse pathological contexts, including infectious diseases, autoimmune disorders, and cancer.

Emerging evidence identifies the basic helix–loop–helix family member E40 (Bhlhe40) as a critical orchestrator of mitochondrial metabolism in CD8^+^ T_RM_ cells [81]. Mechanistically, Bhlhe40 binds to class B E‐box sequences (CACGTG) within mitochondrial genes, amplifying oxidative phosphorylation (OXPHOS) pathways to sustain CD8^+^ T cell energetics [82, 83]. Beyond metabolic regulation, Bhlhe40 modulates epigenetic landscapes and effector functions by controlling cellular acetyl‐CoA pools, governing histone H3K9 and H3K27 acetylation, and driving expression of cytotoxic (IFN‐γ, TNF‐α, granzyme B) and chemotactic (CCL3, CCL4) mediators [84]. The heightened Bhlhe40 expression in T_RM_ versus T_CIRCM_ populations likely underlies its tissue‐specific roles. Tumor microenvironments, characterized by nutrient deprivation (e.g., glucose) and hypoxia [85, 86], may preferentially select for Bhlhe40‐dependent T_RM_ cells. These conditions impose metabolic stress on lymphoid‐derived T cells, favoring adaptations mediated by Bhlhe40. As a stress‐responsive protein, Bhlhe40 exerts an effect on the survival and function of CD8^+^ T_RM_ cells under stressful conditions, thereby promoting differentiation in the direction of tissue residence.

The T‐box transcription factors Eomesodermin (Eomes) and T‐box expressed in T cells (T‐bet) play nonredundant yet complementary roles in CD8^+^ T_RM_ cell differentiation and function. Both factors contain a conserved T‐box DNA‐binding domain essential for transcriptional regulation [64]. Mechanistically, they drive cytotoxic effector function by directly upregulating IFN‐γ, perforin, and granzyme expression, with genetic deletion studies confirming their necessity [87]. During differentiation, T‐bet predominates in effector CD8^+^ T cells while Eomes dominates in effector memory populations [88, 89], suggesting specialized roles in lineage commitment. In T_RM_ cells, these factors coregulate tissue residency programs by balancing retention versus egress molecules. Their dynamic expression patterns and target gene specificity establish them as central regulators of T_RM_ cell development and function. Mature CD8^+^ T_RM_ cells consistently exhibit coordinated downregulation of Eomes and T‐bet across tissues [90]. While complete Eomes silencing occurs during T_RM_ differentiation, residual T‐bet expression persists to maintain IL‐15Rβ (CD122) expression and support long‐term survival [91]. TGF‐β signaling drives this transcriptional reprogramming by suppressing both factors during gut T_RM_ development, with T‐bet deficiency (but not Eomes deficiency) rescuing T_RM_ differentiation in TGF‐βR‐deficient models [92]. These findings reveal a sophisticated regulatory logic: complete loss of Eomes enables T_RM_ commitment, partial T‐bet retention sustains survival pathways, and TGF‐β dynamically balances these two programs to orchestrate T_RM_ development. Eomes demonstrates distinct functional roles in T_RM_ cell maintenance across intestinal compartments, supporting survival in the small intestine but not the colon, primarily through upregulation of the anti‐apoptotic factor Bcl‐2 [93]. Both Eomes and T‐bet serve as crucial mediators between cytokine signaling and T_RM_ cell development, though their functional requirements exhibit significant tissue‐specific variation. Notably, the skin, small intestine, and gut display unique dependencies on these transcription factors during T_RM_ differentiation and maintenance, reflecting adaptation to local microenvironmental cues.

#### 2.3.2 Cytokine Regulation

TGF‐β drives CD8^+^ T_RM_ cell differentiation and tissue retention through coordinated molecular programs. This pleiotropic cytokine promotes CD103 expression (blocked by TGF‐β neutralization) [94, 95], induces Hobit/Blimp‐1 to repress KLF2‐S1PR1‐CCR7‐mediated egress [11, 22], and facilitates T_RM_ accumulation in peripheral tissues like skin. These multilayered mechanisms establish TGF‐β as a master regulator of tissue residency programs. TGF‐β signaling orchestrates T_RM_ cell differentiation through precise modulation of T‐box transcription factors [91]. Notably, Eomes downregulation is indispensable for generating CD103^+^ CD8^+^ T_RM_ populations [96], as evidenced by elevated Eomes levels and decreased CD103^+^ CD69^−^ CD8^+^ T_RM_ cells in TGF‐β‐deficient microenvironments, particularly in hepatic tissues [97]. For epithelial T_RM_ (eT_RM_) cells in barrier sites, TGF‐β signaling is absolutely required. The cytokine's pleiotropic actions are spatially limited by integrin‐dependent activation. In lymph nodes (but not spleen), αVβ8‐expressing DCs activate TGF‐β locally, driving eT_RM_ differentiation [98, 99, 100, 101]. This mechanism resolves the paradox of how a ubiquitously secreted cytokine achieves tissue‐specific outcomes in T_RM_ development. Complementary studies demonstrate that CD103^+^ DCs drive lung T_RM_ cell differentiation through TGF‐β‐dependent mechanisms during antigen cross‐presentation [96]. These findings establish TGF‐β not merely as a facilitator of tissue accumulation, but as a defining regulator of T_RM_ cell identity and function. The dual role of TGF‐β in both localization and phenotypic specification underscores its central position in T_RM_ biology.

The gamma chain‐(γC)‐dependent cytokine IL‐15 plays a critical role in sustaining memory CD8^+^ T cell populations, including CD8^+^ T_RM_ cells, through the promotion of homeostatic proliferation and survival [102, 103, 104]. Notably, IL‐15 supplementation enhances tumor‐specific CD8^+^ T cell functionality [105], supporting its potential as an immunotherapeutic target. Clinical observations further underscore its importance, with elevated IL‐15 levels correlating with increased frequencies of functional tumor‐infiltrating lymphocytes and improved disease‐free survival in cancer patients [106]. IL‐15 sustains CD8^+^ T_RM_ cells through a T‐bet‐Hobit‐Bcl2 axis, with IL‐15R deficiency causing T_RM_ cell attrition [97, 104]. Mechanistically, IL‐15 induces Hobit via T‐bet [22], drives T_EFF_ cell migration/accumulation [107, 108], and upregulates Bcl2 for survival [11], collectively ensuring T_RM_ maintenance.

#### 2.3.3 Metabolic Adaptation

T_RM_ cells maintain a unique metabolic signature characterized by mevalonate‐cholesterol pathway activation, diverging fundamentally from circulating memory subsets. This pathway generates critical nonsterol isoprenoids, including coenzyme Q (CoQ), through branched enzymatic reactions [109], with tissue‐specific variations in pathway activity. SREBP2 drives mevalonate‐cholesterol pathway activation in T_RM_ cells, functioning as a master transcriptional regulator that enhances CoQ production. This metabolic reprogramming sustains mitochondrial oxidative phosphorylation and facilitates T cell accumulation in tumors. Thus, SREBP2 emerges as a central coordinator of T_RM_ cell bioenergetics and tissue surveillance [110]. Pharmacological modulation of mevalonate metabolism controls T_RM_ cell development, where FDFT1 inhibition [111] or PDSS2 activation [112] redirects metabolic flux to boost CoQ production. This mevalonate‐cholesterol‐CoQ axis critically supports T_RM_ cell function by maintaining mitochondrial respiration and enhancing antitumor immunity [109], establishing it as both a metabolic checkpoint and therapeutic target in T_RM_ biology.

Fatty acid (FA) metabolism regulates memory T cell subsets in different ways. T_RM_ cells depend uniquely on exogenous FA uptake for membrane biosynthesis and signaling [111, 113], whereas T_CM_ cells utilize de novo FA synthesis to fuel FAO and OXPHOS [112, 114, 115]. Skin T_RM_ cells specifically upregulate FABP4/5 to optimize exogenous FA utilization, revealing tissue‐specific metabolic specialization that sustains long‐term residency and function [116, 117, 118]. FABPs orchestrate tissue‐specific metabolic programming in T_RM_ cells, with FABP4/5 enabling skin T_RM_ cells to uptake and oxidize exogenous FAs for functional maintenance and recall responses [118, 119]. The tissue microenvironment dictates FABP isoform selection in T_RM_ cells, as demonstrated by distinct expression patterns: liver T_RM_ cells dominantly express FABP1 (with minor FABP4), while small intestinal intraepithelial (SI‐IEL) T_RM_ cells preferentially express FABP1, FABP2, and FABP6—both distinct from the FABP4/FABP5 signature of skin T_RM_ cells [120]. This isoform specialization underpins the tissue‐specific metabolic heterogeneity of T_RM_ cells, optimizing their adaptation to tissue‐unique lipid microenvironments. These findings establish FABPs as key mediators of organ‐adapted immunity and potential targets for immunometabolic intervention [121, 122]. In the skin, the scavenger receptor CD36 enhances exogenous FA uptake in T_RM_ cells through its lipid‐binding hydrophobic domains [123, 124, 125, 126]. However, in the tumor microenvironment (TME), CD36 drives dysfunction through a three‐step mechanism: (1) Redox collapse: Tumor cells outcompete T cells for cystine, depleting glutathione (GSH) and disabling ROS clearance. (2) Aberrant CD36 upregulation: Intracellular glutamate accumulation (a byproduct of impaired cystine import) triggers stress signaling pathways that overexpress CD36. (3) Toxic lipid overload: Uncoupled from FABP‐mediated transport, overexpressed CD36 avidly uptakes pathological oxidized lipids (e.g., oxLDL) from the TME. In the absence of GSH, these lipids accumulate as toxic peroxides, inducing ferroptosis and driving the expression of exhaustion markers (PD‐1, TIM‐3), thus crippling antitumor immunity [127]. In summary, CD36 duality is not intrinsic but reflects a “metabolic checkpoint”: it supports FAO and survival when paired with FABPs and healthy redox balance, but promotes exhaustion and cell death when overwhelmed by oxidized lipids in a GSH‐deficient TME.

## 3 STAND DEFEND: Local Antimicrobial Immunity Driven by CD8^+^ T_RM_

Upon reactivation, CD8^+^ T_RM_ cells deliver rapid cytotoxic effector functions while simultaneously serving as sentinels that recruit circulating immune cells. This dual capacity enables them to provide frontline defense against viral, bacterial, and parasitic pathogens [128, 129].

### 3.1 CD8^+^ T_RM_ in Viral Infection

Viruses constitute the predominant pathogens targeted by CD8^+^ T_RM_ cells. This section details their functional contributions in clinically important viral infections.

#### 3.1.1 CD8^+^ T_RM_ in Hepatitis Virus Infection

Liver inflammation, known as hepatitis, is most commonly caused by six or seven hepatotropic viruses, hepatitis A through G [130]. While hepatitis A virus (HAV) typically induces acute, self‐limiting hepatitis, HBV, HCV, and HDV often establish chronic infections that can progress to cirrhosis and hepatocellular carcinoma [131, 132, 133]. Hepatic CD8^+^ T_RM_ cells play a pivotal role in anti‐HBV immunity, undergoing clonal expansion and long‐term persistence in the liver. These cells mediate antiviral surveillance through IL‐2 and IFN‐γ secretion while targeting diverse HBV epitopes, establishing comprehensive local immune defense [134, 135]. In contrast, during HAV infection, CD69^+^ CD103^−^ T_RM_ cells contribute to liver pathology via IL‐15‐mediated bystander activation and NKG2D‐dependent cytotoxic mechanisms [136]. CD69^+^ CXCR6^+^ T_RM_ cells promote immunopathology in HDV infection through bystander activation and proinflammatory cytokine production, correlating with severe hepatitis outcomes [137]. Notably, HBV‐specific CD8^+^ T_RM_ cells demonstrate dual functionality, serving as critical effectors in HCC antitumor immunity. Their levels of tumor infiltration strongly correlate with improved recurrence‐free survival, maintaining a polyfunctional, nonterminally exhausted phenotype [138, 139]. These findings position virus‐specific CD8^+^ T_RM_ cells as promising prognostic biomarkers for HBV‐related HCC progression.

#### 3.1.2 CD8^+^ T_RM_ in Influenza Infection

Influenza is an acute respiratory illness affecting both mammalian species and domesticated avian populations. The virus originates from zoonotic reservoirs, primarily aquatic birds and bats, and is responsible for recurrent seasonal outbreaks as well as intermittent global pandemics characterized by significant disease burden and mortality rates [140]. Numerous studies highlight that respiratory tract CD8^+^ T_RM_ cells play a critical role in protecting against influenza virus infection [141]. Wu et al. first demonstrated the indispensable role of local tissue‐bound memory CD8^+^ T cells in mediating cross‐protective immunity against the influenza virus [142]. Following viral entry, antigen‐specific CD8^+^ T_RM_ cells undergo rapid reactivation into secondary effector populations. These cells mediate antiviral immunity through coordinated secretion of effector molecules, including IFN‐γ, TNF‐α, perforin, and granzyme B [143, 144, 145]. In the upper respiratory tract, CD8^+^ T_RM_ cells block influenza's spread to the lungs, prevent severe pulmonary disease, and offer long‐term local mucosal protection with unique development traits [146]. Notably, pulmonary CD8^+^ T_RM_ cells localize in bronchus‐associated lymphoid tissue (BALT), eliminating influenza‐infected cells to mediate long‐term local antiviral immunity [147].

Accumulating evidence indicates that pulmonary CD8^+^ T_RM_ cells mitigate the severity of severe acute respiratory syndrome coronavirus 2 (SARS‐CoV‐2) infection. Studies in SARS‐CoV‐2‐infected patients have confirmed the presence of lung CD8^+^ T_RM_ cells, and increased CD8^+^ T_RM_ cell numbers are closely associated with milder disease [148]. These pulmonary CD8^+^ T_RM_ cells combat SARS‐CoV‐2 via IFN‐γ secretion and cytotoxicity, reduce disease severity, and establish long‐lived mucosal memory following viral clearance [149, 150]. Overall, CD8^+^ T cells in SARS‐CoV‐2‐infected patients exhibit enhanced activation, proliferation, and survival without exhaustion and establish a durable CD8^+^ T_RM_ cell population.

#### 3.1.3 CD8^+^ T_RM_ in HIV Infection

Human immunodeficiency virus (HIV), the etiologic agent of acquired immunodeficiency syndrome (AIDS), continues to impose an enormous global disease burden despite remarkable progress in the global response [151]. It is well established that CD8^+^ T cells are required for effective immune control of HIV [152]. Buggert et al. [153, 154] revealed that lymphoid tissue (LT)‐resident HIV‐specific CD8^+^ T_RM_ cells possess unique functional and phenotypic characteristics distinct from their circulating counterparts in peripheral blood. Moreover, these cells are more frequent during natural control of HIV, suggesting that T_RM_ cells may play an important role in limiting HIV replication. The Genital mucosa is the main portal of entry for HIV. Localized in the female genital tract epithelium, CD8^+^ T_RM_ cells exert cytotoxicity via granzyme and perforin, with CD103 mediating retention to defend against HIV [155, 156]. In human gastrointestinal mucosa, CD8^+^ T_RM_ cells comprise CD103^High^ and CD103^Low^ subsets, and HIV‐1 gag‐specific tissue‐resident CD8^+^ T‐cell responses originate predominantly from the CD103^Low^ cells and appear strongest in controllers [157]. Eomes expression in CD8^+^ T_RM_ cells is associated with low CD103 expression and is highest in HIV‐1^+^ participants not receiving antiretroviral therapy (ART) [157, 158]. Notably, HIV‐specific CD8^+^ T_RM_ cells localize to mucosal tissues and lymphoid organs, exerting polyfunctional anti‐HIV effects. Elucidating their protective mechanisms is key to harnessing these potent effector T cells for developing novel HIV reservoir‐targeted therapies.

### 3.2 CD8^+^ T_RM_ in Bacterial Infection

*Listeria monocytogenes* is a foodborne Gram‐positive bacterium that causes listeriosis, a life‐threatening systemic infection characterized by bacteremia and frequently complicated by central nervous system involvement, particularly meningoencephalitis in immunocompromised hosts [159, 160]. CD8^+^ T_RM_ cells contribute to protection against oral infection with the intestinal pathogen *Listeria monocytogenes* [161, 162]. Oral infection induces the mesenteric lymph nodes to initiate an antigen‐specific CD8^+^ T cell response [163, 164]. Memory precursor effector cells (MPECs) undergo rapid accumulation in both the intestinal lamina propria and intraepithelial compartments through selective apoptosis of resident short‐lived effector cells (SLECs) [70, 165]. These MPECs express high levels of CD69 and CD103 and serve as the precursors of intestinal CD8^+^ T_RM_ cells [162, 165, 166]. Collectively, these findings suggest that *Listeria monocytogenes* infection‐induced CD8^+^ T_RM_ rapidly arise from MPECs expressing CD103 and CD69. These precursors seed intestinal tissues early after oral infection, with CD103 expression providing a selective advantage for epithelial accumulation.

### 3.3 CD8^+^ T_RM_ in Parasitic Infection

Malaria, caused in humans by *Plasmodium* parasites (mainly *Plasmodium falciparum* and *Plasmodium vivax*) transmitted through the bite of *Anopheles* mosquitoes, greatly impacts public health and socioeconomic development [167, 168]. In liver‐stage malaria, CD8^+^ T cells play an important role in protection [169, 170]. During malaria infection, intrahepatic CD8^+^ T cells swiftly clear liver‐stage parasites through recognizing parasite epitopes presented on hepatocytes [171]. Notably, CD8^+^ T_RM_ cells are regarded as a key component of the protective immunity against various intracellular pathogens [172, 173]. Irradiated *Plasmodium* sporozoite‐induced liver CD8^+^ T_RM_ cells display a distinct tissue‐specific transcriptome signature: high expression of CXCR3, CXCR6, granzyme B, Ki67, CD69, and T‐bet, and low expression of CD62L, KLRG1, Eomes, and CD127, with no classic exhaustion markers [174]. Liver CD8^+^ T_RM_ cells localize to hepatic sinusoids and patrol along the sinusoidal walls, acting as a front‐line defense against liver‐stage malaria. They rapidly eliminate *Plasmodium*‐infected hepatocytes via robust expression of effector molecules such as IFN‐γ, TNF‐α, and granzyme B [15, 174]. These cells may also contribute positively to responses against other viral and bacterial pathogens that target the liver.

## 4 Ignite Damage: CD8^+^ T_RM_ Cells in Autoimmunity

T_RM_ cells contribute to local immune defense and tissue homeostasis, but they can also drive localized recurrent inflammation [10, 175]. In this section, we sought to investigate the role of T_RM_ cells in diverse autoimmune diseases.

### 4.1 CD8^+^ T_RM_ in Skin‐Autoimmune Diseases

Autoimmune dermatoses, including cutaneous lupus erythematosus (CLE), psoriasis, and vitiligo, involve pathogenic mechanisms wherein distinct T_RM_ cell subsets play key roles, as evidenced by multiple experimental and clinical studies [176].

#### 4.1.1 CD8^+^ T_RM_ in Cutaneous Lupus Erythematosus

Cutaneous lupus erythematosus (CLE) represents an autoimmune‐mediated inflammatory dermatosis that may present as either an isolated condition or, more frequently, as a cutaneous manifestation of systemic lupus erythematosus (SLE) [177, 178]. T lymphocytes constitute the predominant immune population in lesional skin, with additional pathogenic contributions from plasmacytoid dendritic cells (pDCs) and myeloid dendritic cells (mDCs). A positive correlation between disease severity and the frequency of a specific population of CD8^+^ CD103^+^ T_RM_ cells has been identified in patients [37]. Hydroxychloroquine (HCQ) and quinacrine (QC) serve as first‐line systemic oral antimalarials for all cutaneous lupus erythematosus (CLE) subtypes. Notably, elevated frequencies of T_RM_ cells are observed in the skin of antimalarial‐refractory CLE patients. Specifically, CD69^+^ T_RM_ cells were significantly higher in HCQ^+^ QC^−^ nonresponders compared with HCQ^−^ and HCQ^+^ QC^−^ responders [178]. Accordingly, these findings suggest that T_RM_ cells in lesional skin may serve as a key immunological hallmark underlying the persistent inflammation and poor therapeutic response in refractory CLE patients. This finding provides novel insights into the immunopathogenesis of antimalarial‐refractory CLE and identifies promising directions for targeted immunotherapy in this patient subset.

#### 4.1.2 CD8^+^ T_RM_ in Psoriasis

Psoriasis is a chronic, immune‐mediated inflammatory disease that primarily affects the skin but also has systemic implications, affecting the joints, cardiovascular system, and metabolic pathways [179, 180]. A previous study has demonstrated that the marker CD49a identifies two distinct populations of CD103^+^ CD8^+^ T_RM_ cells. In skin from patients with vitiligo, where melanocytes are eradicated locally, CD49a^+^ CD8^+^ T_RM_ cells that constitutively expressed perforin and granzyme B accumulated both in the epidermis and dermis. Conversely, CD49a^−^ CD8^+^ T_RM_ cells from psoriasis lesions predominantly generated IL‐17 responses that promote local inflammation in this skin disease [40]. Kurihara et al.’s [181] study indicates that IL‐17A‐producing CD103^+^ CD8^+^ T_RM_ cells promote psoriasis progression by secreting IL‐17A, correlating with epidermal thickening. Meanwhile, PD‐1^+^ CD103^+^ CD8^+^ T_RM_ cells have been identified as key pathogenic cells driving the progression of psoriasis, with their frequency correlating with disease severity and histopathological changes. These PD‐1^+^ T_RM_ cells possessed a canonical psoriasis‐specific resident memory phenotype characterized by low CD49a expression, along with IL‐23R expression and IL‐17A production, whereas PD‐1^−^ T_RM_ cells preferentially produced IFN‐γ [182].

#### 4.1.3 CD8^+^ T_RM_ in Vitiligo

**FIGURE 3:**
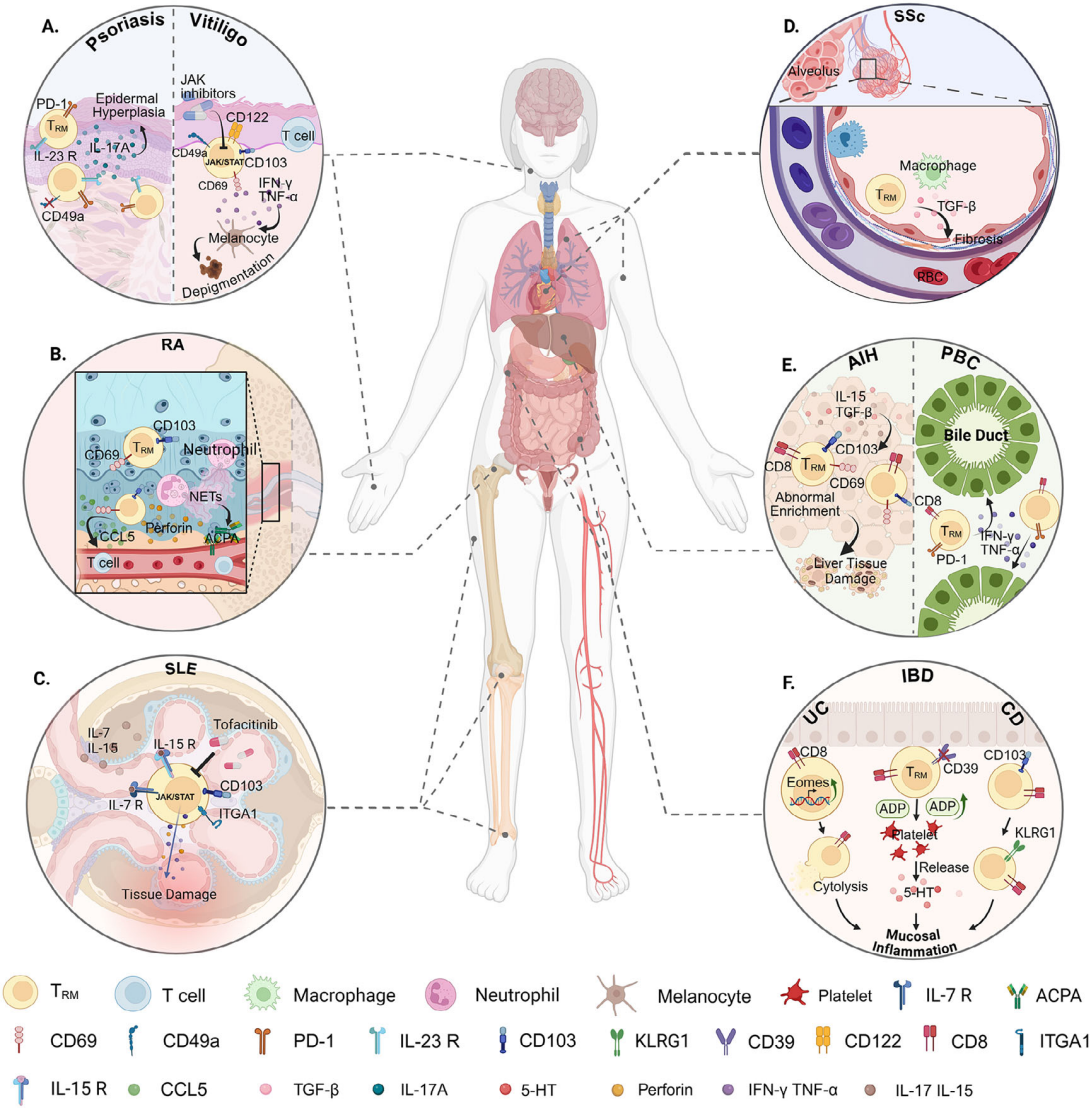
Pathogenic mechanisms of CD8^+^ T_RM_ cells across diverse autoimmune diseases. (A) In psoriasis and vitiligo, skin T_RM_ cells drive epidermal hyperplasia via IL‐17A and induce melanocyte apoptosis via IFN‐γ/TNF‐α. (B) In RA, synovial T_RM_ cells trigger NET formation and subsequent ACPA production to fuel recurrent joint inflammation. (C) In SLE, the IL‐7/15‐JAK/STAT axis maintains T_RM_ effector functions to mediate tissue damage, which can be inhibited by tofacitinib. (D) In SSc, alveolar T_RM_ cells interact with macrophages and secrete TGF‐β to drive pulmonary fibrosis. (E) In AIH and PBC, abnormally enriched hepatic T_RM_ cells mediate extensive tissue damage and exert direct cytotoxicity on bile ducts. (F) In IBD, intestinal T_RM_ cells amplify mucosal inflammation through Eomes‐mediated cytolysis and ADP/5‐HT‐driven pathways.

The clinical feature of vitiligo is the emergence of distinct, patchy white lesions on the skin, a phenotype directly driven by the misdirected cytotoxic activity of CD8^+^ T cells against resident cutaneous melanocytes. Studies of vitiligo have provided important insights into CD8^+^ T cell memory and the establishment of resident memory against self‐antigens [183]. In vitiligo, CD8^+^ T cells persist without overt functional exhaustion. Notably, in patients with melanoma‐associated vitiligo (MAV), clonotypes shared between peripheral blood and skin tissues have been detected for up to 9 years, a finding indicative of T cell resident memory [184]. Furthermore, melanocyte‐specific CD8^+^ T cells are more abundant in the skin than in the peripheral blood, and exhibit a CD103^+^ CD69^+^ CD49a^+^ CD122^+^ T_RM_ cell phenotype, with high levels of IFN‐γ and TNF‐α production (Figure 3A) [38, 40, 185]. The IFNγ‐CXCR3 axis has been established as essential for autoreactive CD8^+^ T cell trafficking in vitiligo pathogenesis across both murine models and human patients, with its activity strongly correlating with disease progression and clinical severity [185, 186]. In addition, JAK inhibitors have been shown to reverse the depigmentation of vitiligo lesions. This effect is mediated through the JAK‐STAT signaling pathway, which critically regulates T_RM_ cell‐effector functions [187]. By blocking IFN‐γ signaling, these inhibitors induce skin repigmentation, demonstrating that T_RM_ cell‐targeting represents a viable therapeutic strategy for vitiligo [187, 188].

### 4.2 CD8^+^ T_RM_ in Rheumatic and Musculoskeletal Autoimmune Diseases

Most rheumatic and musculoskeletal autoimmune diseases can be positioned along a spectrum of disorders [189]. T_RM_ cells establish long‐term residence in nonlymphoid tissues, where they mediate rapid localized immune protection. However, growing evidence indicates these cells also function as critical mediators of chronic inflammation and disease relapse in autoimmune disorders, including rheumatoid arthritis (RA), systemic lupus erythematosus (SLE), and systemic sclerosis (SSc).

#### 4.2.1 CD8^+^ T_RM_ in Rheumatoid Arthritis

Rheumatoid arthritis (RA) is a chronic, inflammatory autoimmune disease associated with autoantibodies targeting various molecules, including modified self‐epitopes [190]. Emerging research indicates that arthritis recurrence is driven by synovial T_RM_ cells, which accumulate in affected joints, persist through clinical remission, and upon antigenic reactivation, precipitate localized disease flares in previously involved joints [191]. CD8^+^ T_RM_ cells have been found to stably reside in the synovial fluid of RA patients and drive localized recurrent inflammation. They display upregulated expression of CD69, CXCR6, and CD49a, accompanied by downregulated expression of S1PR1 and KLF2 [192]. Chang and colleagues identified that T_RM_ cells in human RA are predominantly CD8^+^ and exhibit restricted T cell receptor (TCR) diversity, together with transcriptional signatures indicative of immune activation and cell recruitment, suggesting a pro‐inflammatory role [193]. Additional studies also proved that synovial fluid CD69^+^ CD8^+^ T_RM_ cells can recruit circulating effector T cells by releasing CCL5 and interact with neutrophils to trigger the formation of neutrophil extracellular traps (NETs). Notably, T_RM_ cell‐derived perforin promotes histone citrullination in NETs [192, 194, 195]. These citrullinated proteins serve as autoantigens that elicit the production of anticitrullinated protein antibodies (ACPA) in RA patients, reinforcing their role in sustaining the pathogenic loop underlying disease recurrence [192]. These insights nominate T_RM_ retention or effector programs as potential precision‐targeting strategies to control RA synovitis and joint destruction (Figure 3B).

#### 4.2.2 CD8^+^ T_RM_ in Systemic Lupus Erythematosus

Systemic lupus erythematosus (SLE) is a systemic autoimmune disorder characterized by multiorgan involvement, including the skin, joints, central nervous system, and kidneys [196, 197, 198]. In SLE, up to 50% of patients develop lupus nephritis (LN), and T cells are the most abundant immune cells in biopsies from these patients [199]. Renal T_RM_ cells are more abundant in the inflamed renal tissue of LN patients than in healthy kidneys, and exhibit a ZNF683 (HOBIT)^+^ CD103^+^ ITGA1^+^ KLF2^−^ CD8^+^ T_RM_ cell phenotype, with moderate levels of granzyme K production and a prominent interferon response signature [200]. Renal tissues of LN mice exhibit high expression of IL‐7 and IL‐15, which signal through the JAK/STAT pathway to maintain the stable pool of T_RM_ cells. JAK/STAT signaling is essential for sustaining the effector functions of CD103^+^ CD8^+^ T_RM_ cells, which produce pro‐inflammatory cytokines (TNF‐α, IFN‐γ) and cytotoxic molecules (perforin, granzyme B) to drive renal inflammation and damage (Figure 3C) [37]. To develop T_RM_ cell‐targeting therapeutic strategies, further studies should focus on characterizing renal T_RM_ cells and their microenvironments in different disease conditions.

#### 4.2.3 CD8^+^ T_RM_ in Systemic Sclerosis

Systemic sclerosis (SSc) represents a prototypic immune‐mediated fibrotic disorder, characterized by progressive fibrosis affecting multiple organ systems, including the skin, pulmonary tissue, cardiovascular system, renal parenchyma, and gastrointestinal tract [201, 202, 203]. Emerging evidence implies that aberrantly activated T_RM_ cells not only infiltrate SSc lesions but also directly fuel fibrogenesis by secreting key profibrotic cytokines such as TGF‐β and IL‐13 [204, 205, 206]. TGF‐β is a central mediator of fibrosis, promoting fibroblast proliferation, differentiation into myofibroblasts, and extracellular matrix deposition [207]. In SSc, T_RM_‐derived TGF‐β drives fibroblast activation and tissue fibrosis, accompanied by epigenetic reprogramming of local immune cells [208, 209, 210]. In systemic sclerosis‐associated interstitial lung disease (SSc‐ILD), lung‐resident CD8^+^ T_RM_ cells accumulate significantly and promote pulmonary inflammation and fibrosis by activating T cell receptor and fibrosis‐related signaling pathways, thus contributing to the progression of lung injury [211]. Skin‐resident CD28^−^ CD8^+^ T_RM_ cells promote early‐stage cutaneous fibrosis in systemic sclerosis through two distinct pathways: (1) IL‐13‐mediated paracrine stimulation of collagen production in dermal fibroblasts, and (2) direct perforin/granzyme B‐dependent cytotoxicity targeting both fibroblasts and vascular endothelial cells [212]. Taken together, existing evidence supports that CD8^+^ T_RM_ cells play a pathogenic role in SSc, promoting tissue fibrosis via the secretion of profibrotic mediators (Figure 3D).

### 4.3 CD8^+^ T_RM_ in Digestive System‐Autoimmune Diseases

#### 4.3.1 CD8^+^ T_RM_ in Liver Autoimmune Diseases

Primary biliary cholangitis (PBC) is a cholestatic autoimmune liver disease characterized by an autoreactive T‐cell response against intrahepatic small bile ducts [213]. CD8^+^ T_RM_ cells are the key pathogenic effector cells in PBC. They accumulate in the liver of both PBC patients and experimental models, highly expressing activation‐related molecules, cytotoxic effector molecules, and the immune checkpoint PD‐1, and are closely localized around biliary epithelial cells (BECs). These cells exert strong cytotoxicity to directly induce BEC apoptosis, thereby driving intrahepatic biliary damage, portal inflammation, and liver fibrosis in PBC [214]. Autoimmune hepatitis (AIH) is a severe liver disease that affects children and adults worldwide [215]. Multiple studies have demonstrated an increase in activated CD8^+^ T cells in the liver of patients with AIH [216]. In AIH patients, hepatic CD8^+^ T_RM_ cells exhibiting a CD69^+^ CD103^+^ phenotype demonstrate pathologic expansion, driven by synergistic IL‐15 and TGF‐β signaling (Figure 3E). The accumulation level of these CD8^+^ T_RM_ cells positively correlates with the disease severity of AIH, indicating them as a critical pathogenic subset that mediates hepatic tissue injury in AIH [217].

#### 4.3.2 CD8^+^ T_RM_ in Inflammatory Bowel Disease

Inflammatory bowel disease (IBD), encompassing Crohn's disease (CD) and ulcerative colitis (UC), is a chronic, progressive condition that affects millions of individuals worldwide [218, 219]. In CD, CD103^+^ CD8^+^ T_RM_ cells are elevated in the intestinal lamina propria, whereas KLRG1^+^ CD8^+^ nonresident effector T cells show no increase in inflammatory tissues [220, 221]. In UC, upregulation of key factors such as Eomes in intestinal CD8^+^ T_RM_ cells promotes their conversion into a pathogenic state exhibiting enhanced inflammatory and cytolytic properties, thus mediating mucosal inflammation and playing a central role in the initiation and progression of UC [222]. Furthermore, colonic CD103^+^ CD8^+^ T_RM_ and CD39^+^ CD8^+^ T_RM_ cells were decreased in pediatric IBD compared with controls. The impairment of CD39^+^ CD8^+^ T_RM_ cells leads to impaired purine metabolism, excessive ADP accumulation, subsequent platelet activation and 5‐HT release, and ultimately amplifies intestinal mucosal inflammation [223, 224, 225]. Collectively, these studies support the role of active cytotoxic CD8^+^ T_RM_ cells in both initiation and chronic progression of IBD (Figure 3F).

## 5 KEEP VIGIL: CD8^+^ T_RM_ Cells in Tissue Immunosurveillance and Cancer Immunity

CD8^+^ T_RM_ cells play important roles in controlling and eliminating malignant cells through tumor surveillance and mediating antitumor immunity. By stably residing in peripheral tissues, these nonrecirculating lymphocytes directly recognize and eliminate tumor cells, exerting robust cytotoxicity to constrain tumor initiation, growth, and local invasion.CD8^+^ T_RM_ cells also shape the immune landscape and interact with other immune cells.

### 5.1 CD8^+^ T_RM_ Cells in Cancer Immunosurveillance and Control

Malignancy can be suppressed by the immune system in a process termed immunosurveillance [226]. The mechanisms by which the immune system regulates and shapes cancer progression can be divided into three distinct phases of tumor‐immune interaction: elimination, equilibrium, and escape [227]. CD8^+^ T_RM_ cells critically mediate tumor protection during elimination, demonstrated in melanoma, colorectal, and head/neck cancers [228, 229, 230, 231, 232]. These cells combine tissue‐residency with migratory coordination and employ dual cytotoxic mechanisms: (1) perforin/granzyme‐mediated killing and (2) Fas‐FasL‐induced apoptosis [97, 233, 234]. CD8^+^ T_RM_ cells preferentially utilize CD103‐E‐cadherin binding to enhance tumor cytotoxicity [235]. CD103 stabilizes the immunological synapse, promoting polarized delivery of effector molecules (granzyme B, IFN‐γ) while recruiting CCR5 to facilitate autologous tumor migration [236, 237, 238]. This E‐cadherin‐dependent mechanism is frequently compromised during epithelial‐to‐mesenchymal transition due to loss of tumor E‐cadherin expression [239]. CD8^+^ T_RM_ cells in solid organs (lung, skin, breast) exhibit microenvironment‐dependent plasticity shaped by lifelong environmental exposures and physiological dynamics, directly modulating early immune pressure and immunosurveillance. Their pre‐malignant activity critically influences tumor immunoediting and subsequent immunotherapy outcomes. Two factors come into play: tissue‐specific antigen encounter history and the differential activation thresholds that persistent tissue residency creates.

### 5.2 CD8^+^ T_RM_ Cells Mediate the Amplification of Antitumor Immunity

In addition to their potent cytotoxic abilities, CD8^+^ T_RM_ cells play a critical role in shaping the immune landscape by secreting significant amounts of effector molecules, including IFN‐γ and TNF‐α. Reactivated T_RM_ cells can broadcast the detection of pathogen‐associated peptides to other cell types through cytokines. DCs are principal messengers between the innate and adaptive immune systems [240, 241]. Menares et al. demonstrate that skin T_RM_ cell activation promotes maturation and trafficking to draining lymph nodes of migratory dermal DCs. Detection of broadly conserved antigen‐associated molecular patterns instructs DCs maturation, which in turn initiates T cell activation [240, 241]. This work highlights the ability of T_RM_ cells to cross‐talk with DCs and orchestrate the broadening of anti‐tumor CTL immunity [46]. IFN‐γ produced by T_RM_ induces an innate‐like alarm state characterized by the production of chemokines in the tissue and the recruitment of T_CM_ cells. CD8^+^ T_RM_ cell‐derived IL‐2 promotes upregulation of granzyme B in Natural Killer (NK) cells, further boosting the anti‐tumor response [242]. Previous work has shown that CD8^+^ T_RM_ cells rapidly re‐express IFN‐γ after local antigen rechallenge. T_RM_ cells also secrete TNF‐α to mature local DCs and IL‐2 to activate NK cells upon antigen stimulation [242, 243, 244]. While requiring antigen as an initial trigger, T_RM_ cells upregulate broad‐spectrum defense genes independently of ongoing antigen recognition, mirroring effector CD4^+^ T cell mechanisms [19]. CD8^+^ T_RM_ cells, while initially activated through TCR‐dependent antigen recognition, upregulate broadly reactive genes that enable antigen‐nonspecific responses, functionally positioning them as a bridge between adaptive and innate immunity where the TCR operates analogously to innate immune Toll‐like receptors [129].

## 6 Preclinical and Clinical Trials Targeting CD8^+^ T_RM_ Cells in Different Diseases

Capitalizing on the unique immunological properties of CD8^+^ T_RM_ cells—including tissue‐resident persistence, long‐term protective memory, and rapid recall responses—both preclinical research and clinical trials targeting these cells are being actively pursued across multiple disease indications. Beyond vaccinology approaches aimed at T_RM_ cell generation, current therapeutic strategies fall into four principal categories: (1) monoclonal antibody‐based targeting, (2) immune checkpoint modulation, (3) cytokine pathway engineering, and (4) adoptive cellular therapies—all designed to either harness or regulate CD8^+^ T_RM_ cell functionality for enhanced therapeutic efficacy.

### 6.1 Therapies Targeting CD8^+^ T_RM_ Cells in Infectious Diseases

Given the ability of CD8^+^ T_RM_ cells to confer long‐term immune protection and their distinctive immunological memory characteristics, researchers have been developing T_RM_ cell‐targeted vaccines to elicit these cells across diverse tissues and mucosal sites. This notion is supported by murine and human vaccination studies, which have demonstrated that mucosal immunization effectively elicits T_RM_ cells at mucosal sites against pathogens including influenza [245], SARS‐CoV‐2 [246], respiratory syncytial virus (RSV) [247], and HIV [248]. Several types of vaccine formulations have been tested for their capacity to trigger T_RM_ cells, and most have shown excellent efficacy in animal studies. In influenza vaccine development, strategies to generate respiratory tract CD8^+^ T_RM_ cells employ two principal approaches: (1) direct mucosal antigen delivery to exploit tissue‐specific differentiation signals, or (2) systemic‐prime/mucosal‐boost regimens that leverage compartmentalized immune programming for optimal T_RM_ cell development [249, 250, 251]. Key viral vectors, such as adenovirus, modified vaccinia Ankara (MVA), and murine cytomegalovirus (MCMV), serve as the core platforms for achieving this efficient intracellular antigen delivery [249, 252, 253]. A single intranasal dose of the chimpanzee adenovirus (ChAd)‐vectored SARS‐CoV‐2 spike (S) vaccine in human ACE2‐transgenic mice induced: (1) potent neutralizing antibodies, (2) systemic and mucosal IgA responses, and (3) antigen‐specific T cell immunity. Crucially, the generation of pulmonary SARS‐CoV‐2‐specific CD8^+^ T_RM_ cells emerged as a key protective correlate against both upper and lower respiratory tract viral challenge [246, 254, 255].

In the murine lungs, Morabito KM. et al. demonstrated that intranasal immunization with RSV M protein‐expressing MCMV elicits robust and long‐lived tissue‐resident effector/memory CD8^+^ T cells, mediating accelerated RSV clearance [247, 256]. Studies on HIV‐specific T_RM_ vaccination strategies have focused on lodging CD8^+^ T_RM_ cells in the genital and respiratory tract [251, 257]. Vaccines that establish CD8^+^ T_RM_ cells in the cervicovaginal mucosa hold great promise for eliciting effective immunity against sexually transmitted HIV, and this can be achieved via combined intranasal and intravaginal mucosal immunization with recombinant influenza‐HIV vectors [248]. As liver CD8^+^ T_RM_ cells have recently been shown to control liver‐stage *Plasmodium* infections, many studies are employing vaccines to induce liver T_RM_ cells to prevent malaria [258]. Ribosomal protein L6 (RPL6) is an ideal antigen for liver CD8^+^ T_RM_ cell‐mediated immunity, and prime‐and‐trap vaccination targeting this protein confers effective protection against malaria [259]. Messenger RNA (mRNA)‐based vaccine strategies induce liver CD8^+^ T_RM_ cells, thereby establishing tissue‐specific immunity to prevent malaria at the critical pre‐erythrocytic liver stage [35]. Collectively, these translational studies showcase a range of immunotherapeutic interventions to target CD8^+^ T_RM_ cells in infectious diseases, with a common requirement of engaging T_RM_ cell‐intrinsic receptors or cytokines to facilitate optimal antimicrobial immunity (Table 1).

**TABLE 1:** Preclinical and clinical trials targeting CD8^+^ T_RM_ cells in different inflammatory diseases.

Disease Type	Medications/Methods	Mechanism	Phase	References
**Autoimmune disease**				
CLE	Secukinumab (anti‐IL‐17A)	Neutralizes pathogenic IL‐17A and blocks its locally driven inflammatory pathways in the skin	Phase II	NCT03866317
Psoriasis	Risankizumab (anti‐IL‐23)	IL‐23 blockade downregulates keratinocyte‐derived IL‐15/IL‐7, reducing CD8^+^ T_RM_ cell survival and function	Phase IV	NCT04433442
	Guselkumab (anti‐IL‐23)	Inhibits IL‐23p19, reducing CD8^+^ T_RM_ cells in skin and blood while preserving Treg cells, thereby suppressing IL‐23‐driven inflammation	Phase IV	NCT04080648
	Secukinumab (anti‐IL‐17A)	Neutralizes IL‐17A, disrupting the IL‐23/IL‐17 feedback loop and suppressing CD8^+^ T_RM_‐driven inflammation.	Phase II	NCT03131570
	Risankizumab (anti‐IL‐23 inhibitors)	Decreases T_RM_ cells and prevents their repopulation, exerting a disease‐modifying effect to prolong DFR	Phase III	[270]
Vitiligo	AMG 714 (anti‐IL‐15)	Blocks IL‐15 signaling, disrupting keratinocyte‐mediated T_RM_ cell survival and effector function, thereby reducing CD8^+^ T_RM_‐mediated melanocyte destruction	Phase II	NCT04338581
	Tofacitinib, Ruxolitinib (JAK inhibitors)	Inhibits the IFN‐γ signaling pathway to block T cell function without depleting established skin T_RM_ cells	Preclinical	[188]
RA	anti‐IL‐15Rα and anti‐CD122 antibodies	Blocks IL‐15 trans‐presentation by synovial fibroblasts/macrophages, preventing T_RM_ activation, perforin upregulation, and subsequent NETosis	Preclinical	[192]
SSc	Romilkimab (anti‐IL‐4, IL‐13)	Neutralizes IL‐4/IL‐13 to interrupt the T_RM ‐_driven cutaneous fibrotic and inflammatory responses in SSc skin lesions	Phase II	[208]
	Anti‐IL‐13 neutralizing antibody	Targets high IL‐13 production by skin CD8^+^ CD28^−^ T_RM_ cells to prevent direct induction of fibroblast collagen production	Preclinical	[212]
PBC	CAR‐T	Selectively depletes pathogenic PD‐1‐expressing hepatic CD8^+^ T_RM_ cells, alleviating autoimmune bile duct damage	Preclinical	[214]
IBD	Dipyridamole (PDE inhibitor)	Elevates cAMP to restore colonic CD39^+^ CD8^+^ T_RM_ cells, suppresses platelet aggregation and TNF‐α, ameliorating pediatric colitis	Preclinical	[223]
**Infectious disease**				
Influenza	Live attenuated influenza vaccine	Intranasal LAIV induces lung virus‐specific CD8^+^ T_RM_ cells mediating heterosubtypic protection independent of circulating immunity	Preclinical	[245]
	Subunit vaccine	Induces and maintains NP‐specific CD8^+^ T_RM_ cells in the lung via limited viral replication during primary infection, conferring cross‐protection	Preclinical	[250]
	Viral vector vaccine	Activates M1‐specific CD8^+^ T cells in human nasopharyngeal lymphoid tissue, expanding the T_RM_ pool for rapid degranulation upon challenge	Preclinical	[253]
SARS‐CoV‐2	Viral vector vaccine	Induces lung CD103^+^ CD69^+^ CD8^+^ T_RM_ cells, providing superior protection against SARS‐CoV‐2 and potentially achieving sterilizing immunity	Preclinical	[246]
RSV	Viral vector vaccine	Intranasal immunization induces lung CD8^+^ T_RM_ cells, resulting in accelerated viral clearance upon challenge	Preclinical	[247]
	Subunit vaccine	Induces lung M/M2‐specific CD8^+^ T_RM_ cells, significantly reducing viral load upon RSV challenge	Preclinical	[256]
HIV	Viral vector vaccine	Induces HIV‐specific CD8^+^ T_RM_ cells in vaginal epithelium; upon antigen re‐exposure, upregulates VCAM‐1 and recruits peripheral immune cells	Preclinical	[248]
	Live‐attenuated vaccine	Persistent SIVΔ*nef* replication drives immune maturation: induces vaginal CD8^+^ T_RM_ cells to block SIV transmission and dissemination	Preclinical	[257]
Malaria	mRNA vaccine	Induces the expansion of liver CD8^+^ T_RM_ cells via NKT cell help, enabling local recognition and elimination of Plasmodium parasites in the liver	Preclinical	[35]
	DNA vaccine	DNA prime induces high‐frequency CSP‐specific CD8^+^ T cells; RAS recruits them to the liver to differentiate into T_RM_ cells, providing complete protection	Preclinical	[258]

*Note*: Data sources: https://clinicaltrials.gov.

Abbreviations: CSP, circumsporozoite protein; DFR, drug‐free remission; LAIV, live‐attenuated influenza vaccine; M/M1/M2, matrix protein/matrix protein 1/matrix protein 2; NETosis, neutrophil extracellular traps; NP, nucleoprotein; PDE, phosphodiesterase; RAS, radiation‐attenuated sporozoites; SIV, Simian immunodeficiency virus; SIVΔnef, Simian immunodeficiency virus with a deletion in the Nef gene; Treg cells, regulatory T cells; VCAM‐1, vascular cell adhesion molecule 1.

### 6.2 Therapies Targeting CD8^+^ T_RM_ Cells in Autoimmune Diseases

Previous studies have demonstrated that CD8^+^ T_RM_ cells, while serving as critical sentinels to protect the host against recurrent pathogen invasion, also act as key pathogenic mediators in the initiation, chronicity, and relapse of autoimmune diseases by driving persistent local inflammation and tissue damage. This dual role highlights the necessity of targeted inhibition of pathogenic CD8^+^ T_RM_ cells and their pro‐inflammatory functions as a promising therapeutic strategy for autoimmune disorders. Drug repurposing enables the feasible application of therapeutic antibodies and small‐molecule inhibitors that target the effector cytokines or receptors of CD8^+^ T_RM_ cells. IL‐15 signaling is a critical mediator of the survival and proliferation of CD8^+^ T_RM_ cells, with this cytokine primarily triggering the activation of downstream effectors through the JAK‐STAT pathway [69, 108]. The blockade of CD122, a subunit of the IL‐15 receptor, in a murine vitiligo model demonstrated that short‐term treatment impaired T_RM_ cell effector function via reduced IFN‐γ secretion, and long‐term treatment depletes CD8^+^ T_RM_ cells from skin lesions [38, 260]. Tofacitinib, an inhibitor of JAK1 and JAK3, impaired the survival of renal T_RM_ cells and is effective in the treatment and prevention of LN in MRL/lpr lupus mice, shedding new light on the development of JAK inhibitors as therapeutic strategies for lupus nephritis [37, 261, 262]. In RA, intra‐articular diphtheria toxin injection depletes synovial CD8^+^ T_RM_ cells locally (without affecting circulating T cells), potently suppressing RA flare, T_RM_ expansion, and myeloid cell recruitment [193].

Targeting and disrupting the adhesion, homing, and retention signals of CD8^+^ T_RM_ precursors via therapeutic anti‐trafficking agents has emerged as a major direction for clinical translation in immune‐mediated intestinal disorders. CD8^+^ T cell trafficking from the spleen to intestinal tissues is regulated through suppression of integrin α4β7 expression. This gut‐homing receptor mediates lymphocyte recruitment by binding mucosal addressin cell adhesion molecule‐1 (MAdCAM‐1), selectively expressed on vascular endothelial cells within the intestinal lamina propria [18, 95, 263].

A phase III trial in IBD patients was completed, where the anti‐β7 integrin antibody etrolizumab was administered. Etrolizumab binds the β7 integrin, which can form heterodimers with the αE (CD103) or α4 (Itga4) integrin chain, thus affecting the binding of T cells to E‐cadherin and/or MadCAM‐1, respectively [264]. This agent has been evaluated in a completed Phase III clinical trial for IBD, and it binds to the β7 integrin subunit that heterodimerizes with the αE (CD103) and α4 (Itga4) integrin chains, thereby interfering with T cell adhesion to E‐cadherin and MAdCAM‐1, respectively [265]. Likewise, vedolizumab (anti‐α4β7) and ontamalimab (anti‐MAdCAM1) block T cell homing to the gut, effectively reducing the recruitment and seeding of circulating T_RM_ precursors, thereby limiting the replenishment of pathogenic T_RM_ cells in the inflamed gut [95, 266, 267]. CD103 serves as a defining marker for CD8^+^ T_RM_ cells. In a mouse model of Sjögren's syndrome (pSS), intraglandular administration of anti‐CD103 monoclonal antibody reduced glandular damage and improved salivary flow [268]. Another therapeutic approach under active investigation involves modulating the S1P/S1PR1 pathway, given that S1P downregulation is key to intestinal T_RM_ establishment [36, 47, 269]. Ozanimod, a selective S1PR1 modulator, blocks the gut homing of T_RM_ precursor cells, thereby reducing T_RM_ cell generation and potentially sustaining patients with UC in clinical remission [36, 269]. Additionally, PD‐1^+^ CD8^+^ T_RM_ cells mediate primary biliary cholangitis in mice, and PD‐1‐targeted CAR‐T therapy specifically depletes these pathogenic cells to ameliorate cholangitis with favorable safety, providing a novel precision therapeutic approach for PBC [214]. Given the existing therapeutic strategies targeting CD8^+^ T_RM_ cells in autoimmune diseases, precise inhibition of these pathogenic cells can be achieved through multiple feasible approaches. These include drug repurposing, targeting their survival and proliferation signals, interfering with T_RM_ homing and retention mechanisms, or specifically depleting pathogenic subsets—all of which provide viable therapeutic options for the treatment of related autoimmune disorders (Table 1).

### 6.3 CD8^+^ T_RM_ Cells: Correlations With Clinical Outcomes and Functions in Tumor Immunotherapy

CD8^+^ T_RM_ cells are closely correlated with clinical prognoses and have emerged as pivotal regulators of cancer immunotherapy. Here, we summarize the feasible utilization of these cells in predicting clinical outcomes and optimizing cancer immunotherapies.

#### 6.3.1 Relevance of CD8^+^ T_RM_ Cells in Clinical Outcome

Clinical studies across more than 10 cancer types (breast, lung, colorectal, esophageal, endometrial, ovarian, cervical, bladder, melanoma, liver) demonstrate that CD8^+^ T_RM_ cell infiltration consistently correlates with enhanced antitumor immunity and improved patient survival (Table 2) [271, 272, 273, 274, 275, 276, 277, 278, 279, 280], positioning these cells as key prognostic biomarkers and therapeutic targets. CD8^+^ T_RM_ cells in tumors frequently upregulate exhaustion markers (PD‐1, CTLA‐4, TIM‐3, TIGIT, and LAG‐3) [59, 271, 281, 282, 283, 284, 285], creating a functional dichotomy where their antitumor potential may be constrained by immune checkpoint pathways, potentially limiting immunotherapy efficacy.

**TABLE 2:** Clinical relevance of CD8^+^ T_RM_ in cancer patients.

Cancer type	T_RM_ phenotype	Inhibitory receptors	Clinical response	Prognostic value	References
Breast cancer	CD8^+^, CD103^+^, CD69^+^	PD‐1, CTLA‐4, LAG‐3	The CD8^+^ T_RM_ gene signature is positively correlated with pCR and survival outcomes (distant DFS and OS) in TNBC patients treated with immune checkpoint inhibitors	Positive	[28]
	CD8^+^, CD103^+^, CD69^+^	PD‐1, CTLA‐4, LAG‐3, TIM‐3, TIGIT	The T_RM_ phenotype of CD8^+^ T cells, identified through scRNA‐seq, is significantly associated with improved survival outcomes in TNBC	Positive	[233]
	CD8^+^, CD103^+^, CD69^+^	/	The density of CD103^+^ CD8^+^ T_RM_ cells within cancer islands is positively correlated with RFS, improving RFS in breast cancer patients	Positive	[273]
Lung cancer	CD8^+^, CD103^+^, CD69^+^, CD39^+^, CD49a^+^	PD‐1, 4‐1BB, TIM‐3	The frequency of PD‐1^+^ TIM‐3^+^ T_RM_ cells correlates positively with response to anti‐PD‐1 therapy in lung cancer patients	Positive	[274]
	CD103^+^, CD69^+^, CXCR6	PD‐1	Higher intraepithelial CD103^+^ CD8^+^ T cell infiltration correlates with improved OS in NSCLC patients	Positive	[294]
Colorectal cancer	CD8^+^, CD103^+^	PD‐1, CTLA‐4	The degree of infiltration level of DP CD8^+^ T_RM_ cells is positively correlated with the prognosis of CRC patients, suggesting they possess stronger anti‐tumor activity	Positive	[29]
Endometrial cancer	CD8^+^, CD103^+^	PD‐1, TIGIT	In endometrial cancer patients, a high proportion of CD8^+^ T_RM_ cells co‐expressing PD‐1 and TIGIT is associated with poor prognosis, suggesting that it may be a potential target for immunotherapy	Negative	[30]
Ovarian cancer	CD8^+^, CD103^+^, TIA‐1	PD‐1	The presence of CD103^+^ TILs is strongly associated with increased DSS in HGSC and MOC	Positive	[275]
	CD8^+^, CD103^+^	PD‐1	The presence of PD‐1^+^ TILs is strongly associated with increased DSS in HGSC	Positive	[276]
	CD103^+^	/	The number of CD103^+^cells is related to the improvement of DSS in patients treated with PS	Positive	[277]
	CD103^+^	PD‐1	High CD103^+^ TIL density is positively correlated with improved DSS in HGSC patients treated with primary PS	Positive	[31]
Cervical cancer	CD8^+^, CD103^+^	PD‐1	High infiltration level of CD103^+^ TILs is associated with improved DSS and DFS in cervical cancer patients, particularly in those receiving radiotherapy	Positive	[32]
Bladder cancer	CD8^+^, CD103^+^	/	High infiltration of CD103^+^ CD8^+^ T_RM_ cells is positively correlated with improved OS and better response to PD‐L1 blockade and ACT in MIBC patients	Positive	[33]
	CD103^+^	PD‐1	A higher proportion of tumor‐infiltrating T_RM_ correlates with less advanced tumor stage (stage I vs. ≥ stage II) in bladder cancer patients	Positive	[278]
Melanoma	CD8^+^, CD103^+^, CD69^+^	PD‐1	Higher expression of VLA‐1 on circulating Melan‐A‐specific CD8^+^ T cells is positively correlated with longer OS and DFS in melanoma patients	Positive	[41]
	CD103^+^, CD69^+^, CD49a^+^	PD‐1	The CD8^+^ T_RM_ gene signature correlates with improved survival and enhanced responses to immunotherapy	Positive	[46]
	CD103^+^, CD69^+^, CD49a^+^	PD‐1, CTLA‐4, LAG‐3, TIGIT	The 22‐gene T_RM_ risk score is negatively correlated with patient OS, with high‐risk scores associated with shorter survival	Positive	[279]
HCC	CD8^+^, CD103^+^	PD‐1	Higher infiltration of CD8^+^ T_RM_ cells and elevated JAML expression in tumors correlate with improved OS in HCC patients	Positive	[280]
Gastric cancer	CD103^+^, CD69^+^, CD39^+^	PD‐1, TIGIT	The presence of CD103^+^ CD8^+^ T_RM_ cells is associated with better prognosis in gastric adenocarcinoma patients, and their higher density correlates with improved survival	Positive	[295]
Head and neck cancer	CD8^+^, CD103^+^, CD69^+^	PD‐1, CTLA‐4, TIM‐3	Higher frequencies of CD103^+^ CD39^+^ CD8^+^ TILs are associated with better OS in head and neck cancer patients	Positive	[234]
ESCC	CD8^+^, CD103^+^, CD69^+^	PD‐1, CTLA‐4, TIM‐3	The density of CD103^+^ CD8^+^ TILs in tumor regions is positively associated with improved OS in ESCC patients	Positive	[296]
cSCC	CD103^+^, CD69^+^	PD‐1, CTLA‐4, CD39, IL‐10	High frequencies of CD103^+^ CD8^+^ T_RM_ in primary cutaneous squamous cell carcinoma are significantly associated with increased metastasis risk and reduced DSS	Negative	[286]
ccRCC	CD103^+^, CD69^+^	/	High CD103^+^ T‐cell density is significantly correlated with poor OS in ccRCC patients and increased risk of metastasis development	Negative	[287]

Abbreviations: ACT, adjuvant chemotherapy; ccRCC, clear cell renal cell carcinoma; cSCC, cutaneous squamous cell carcinoma; DFS, disease‐free survival; DP, double positive; DSS, disease‐specific survival; ESCC, esophageal squamous cell carcinoma; HGSC, high‐grade serous ovarian cancer; MIBC, muscle‐invasive bladder cancer; MOC, mucinous ovarian cancer; OS, overall survival; pCR, pathological complete response; PS, primary surgery; RFS, relapse‐free survival; TIL, tumor‐infiltrating lymphocyte; TNBC, triple‐negative breast cancer; VLA‐1, very late antigen‐1.

However, Lai et al. and Sanders et al. demonstrated that CD103^+^ CD8^+^ T_RM_ cells correlate with immunosuppression, tumor progression, and metastasis in cutaneous squamous cell carcinoma (cSCC) and clear cell renal cell carcinoma (ccRCC), predicting a poor prognosis [286, 287]. CD8^+^ CD103^+^ T_RM_ cells had a regulatory and exhausted phenotype characterized by IL‐10 secretion and high expression of PD‐1, CTLA‐4, and CD39, thereby inhibiting CD8^+^ T cell proliferation and facilitating tumor growth [286, 288, 289, 290]. In cSCC, the high expression of TGF‐β, ultraviolet radiation‐induced tissue damage, and increased Treg cells further increase inhibitory CD8^+^ CD103^+^ T_RM_ cells, rendering this subset a key mediator of immunosuppression in this malignancy [291, 292, 293]. Additionally, in preclinical models, tumor‐specific or bystander T_RM_ cells present before tumor onset boosted immune cell recruitment, causing tumor immune evasion through loss of MHC class I protein expression and resistance to immune checkpoint inhibitors [294]. Taken together, CD8^+^ T_RM_ cells exert dichotomous roles in tumor progression: the majority of these cells correlate with favorable clinical outcomes and serve as a positive prognostic factor for cancer patients, while a small subset is associated with poor prognosis by facilitating tumor development and immune escape.

#### 6.3.2 Engineering CD8^+^ T_RM_ Cell Responses as Cancer Therapies

Given that T_RM_ cells in most tumors express exhaustion markers such as PD‐1, they respond to ICIs early on and are key targets during initial treatment [272, 297, 298]. PD‐1 blockade enhances T_RM_‐mediated antitumor immunity by expanding functional T_RM_ pools [298], driving T_CM_‐to‐T_RM_ differentiation [299], and upregulating tissue‐residency genes [233]. T_RM_‐rich tumors correlate with superior ICI responses [272], motivating combination strategies targeting PD‐1/CTLA‐4/4‐1BB/TIGIT [300]. For gastrointestinal cancers, 4‐1BB agonism synergizes with PD‐1 blockade to boost T_RM_ cytotoxicity and proliferation [301, 302, 303]. TIGIT is a co‐inhibitory receptor expressed on CD8^+^ T cells [304]. Dual blockade of TIGIT and PD‐1/PD‐L1 synergistically enhances antitumor immunity across multiple cancers [305, 306, 307]. In endometrial cancer, PD‐1^+^ TIGIT^+^ T_RM_ cells exhibit an exhausted phenotype (impaired cytotoxicity but increased proliferation). Dual PD‐1/TIGIT blockade restores their function better than either alone [30, 308]. T_RM_ cells accumulate postchemoradiotherapy, correlating with improved prognosis in chemotherapy‐treated patients [309, 310]. High T_RM_ infiltration predicts favorable ICB response across cancers [27, 275, 311], while pre‐existing cytotoxic T_RM_ cells enhance pembrolizumab efficacy in head and neck cancer [312]. Clinically, nivolumab plus neoadjuvant chemotherapy boosts survival in gastric cancer (Phase III) [313], underscoring T_RM_ cells’ therapeutic relevance. Targeted blockade of inhibitory receptors on CD8^+^ T_RM_ cells provides a mechanistic rationale for combination immunotherapy, leveraging their dual role as potent cytotoxic effectors and tissue‐persistent immune sentinels to enhance clinical outcomes.

CD8^+^ T_RM_ cells are prime targets for cancer vaccines due to their frontline tumor localization and cytotoxic potency [314]. Infectious disease research has informed vaccine strategies, with prime‐boost and mucosal delivery approaches generating tumor‐specific T_RM_ populations at mucosal sites [315, 316, 317]. For instance, intranasal or intramuscular STxB‐E7 peptide vaccination induces lung T_RM_ cells that suppress tumor growth [232, 318, 319]. While traditional antipathogen prime‐boost regimens often use live virus or DNA vaccines [319], recent applications combine intramuscular DNA priming with intranasal boosting to generate lung T_RM_ cells to prevent lung metastases [317, 320]. Intramuscular immunization primarily generates circulating T cells, with T_CM_ cells serving as key precursors for subsequent lung T_RM_ differentiation [187, 188, 189, 190]. The CXCR6‐CXCL16 axis critically mediates CD8^+^ T_RM_ cell recruitment to lung mucosa postvaccination, enabling tumor control [321]. Current strategies to enhance intratumoral T_RM_ presence include: optimized vaccine delivery systems, heterologous viral vectors with conserved antigens, and “prime‐and‐pull” immunization [68, 322]. “Prime‐and‐pull” immunization first uses conventional vaccination to elicit systemic T‐cell responses (prime). Then, a nonspecific signal or local chemokine “pulls” the T cells generated by prime immunization into the target peripheral tissue, where they differentiate into T_RM_ cells [323, 324]. Prior study found that the intranasal delivery of zymosan could draw effector CD8^+^ T cells into the lung and effectively drive their differentiation into lung T_RM_ cells [325]. Adjuvant development further potentiates these vaccine strategies [326]. Lai JCY et al. demonstrated that topical CpG oligodeoxynucleotides (CpG ODN), a TLR9 agonist, generate skin T_RM_ cells against B16‐OVA melanoma in mice, with Ag‐specific CD8^+^ T cell induction requiring TLR9 on hematopoietic cells and partially on stromal cells [327], highlighting a novel T_RM_ vaccine‐enhancing strategy.

Emerging work highlights that stem‐like memory T (T_SCM_) properties enhance CAR‐T efficacy [328, 329, 330], prompting the development of tissue‐resident memory CAR‐T (CAR‐T_RM_) cells, which combine tissue‐residency advantages with stem‐like durability. Jung et al. revealed that TGF‐β/IL‐2 priming during CAR‐T manufacturing enhances stemness and tissue‐residency traits via KLF2 downregulation [331, 332, 333]. Paradoxically, while TGF‐β suppresses CTL surveillance in tumors [334], its ex vivo use may reprogram CAR‐T cells to overcome TGF‐β‐rich immunosuppressive microenvironments [335, 336]. These protocol modifications offer clinically translatable strategies to enhance CAR‐T efficacy against solid tumors [337].

IL‐15 is essential for T_RM_ cell differentiation and maintenance. Cytokine‐loaded nanogels can deliver IL‐15 directly to the tumor site, helping T_RM_ cells take root and stick around [108, 338, 339]. TGF‐β presents a therapeutic paradox in cancer—essential for T_RM_ cell formation but immunosuppressive in the TME. While blocking TGF‐β signaling in CD8^+^ T_RM_ cells is challenging due to its dual roles [340], future research may identify downstream targets specific to T_RM_ differentiation, enabling selective modulation to uncouple T_RM_ promotion from immune suppression. Pei et al. [37, 341, 342, 343, 344] identified BFAR as a key suppressor of T_RM_ cell generation in aged CD8^+^ T cells, where it inhibits JAK2‐STAT1 signaling via JAK2 deubiquitination. Genetic (*Bfar* knockout) or pharmacological (iBFAR2) blockade of BFAR enhanced T_RM_ formation and restored antitumor immunity, offering a therapeutic strategy for elderly patients or anti‐PD‐1‐resistant cases [344]. Their findings highlight the potential of iBFAR2 to restore antitumor activity in older patients or those resistant to anti‐PD‐1 therapy. Moreover, T_RM_ cells compete with tumors for glucose, amino acids, and FFAs while expressing high FABP4/5 levels. Targeting this metabolic axis via PPAR agonists enhances FFA catabolism, thereby boosting T_RM_‐mediated antitumor immunity and anti‐PD‐1 efficacy [345, 346]. And checkpoint blockade upregulates FABP4/5 to increase lipid uptake and T_RM_ survival (Table 3) [295, 347].

**TABLE 3:** Role of T_RM_ in cancer immunotherapy.

Treatment	specific treatment	Tumor types	Related mechanisms	References
Cancer vaccine therapy	CpG ODN adjuvanted vaccine	Melanoma	Topical CpG ODN activates TLR9 on hematopoietic cells to promote antigen‐specific T cell recruitment and residency in skin via local cytokine modulation	[327]
	CpG‐OVA vaccine	Melanoma	Induces skin CD103^+^ CD8^+^ T_RM_ cells (local and distal) resistant to antibody depletion; protects against melanoma independently of circulating CD8^+^ T cells	[231]
	Intranasal vaccination, anti‐TGF‐β mAb	Head and neck cancer	Mucosal vaccination induces TGF‐β‐dependent T_RM_ cells that persist locally, produce IFN‐γ, and mediate tumor control independently of circulating T cells	[232]
	HPV E6/E7‐targeted therapeutic vaccination^+^ radiotherapy	Cervical cancer	CD103^+^ CD8^+^ T cells are recruited and activated by HPV E6/E7‐targeted vaccination combined with radiotherapy, leading to enhanced tumor infiltration and cytotoxicity	[32]
ICB therapy	Anti–PD‐1 mAb (nivolumab, pembrolizumab)	Melanoma	CD103^+^ tumor‐resident CD8^+^ T cells enhance anti‐tumor immunity through local cytokine production and cytotoxic activity, correlating with improved survival outcomes	[272]
	Adoptive T cell transfer, anti–PD‐1 mb	Melanoma	Anti‐PD‐1 therapy enhances T_RM_ cells differentiation from circulating memory T cells and increases tumor infiltration by blocking PD‐1‐mediated suppression	[230]
CAR‐T_RM_ cell therapy	Anti–TGF‐β mAb^+^ radiotherapy	Colorectal cancer	Tumor‐resident T cells survive radiation via TGF‐β‐mediated reprogramming into a radioresistant state resembling T_RM_, enhancing IFN‐γ production and tumor control	[331]
Targeted T_RM_ therapy	Adoptive T cell transfer	Melanoma	Adoptive transfer of Runx3 gene‐modified CD8^+^ T cells can significantly enhance the differentiation and antitumor function of T_RM_ cells	[21]
	Treg depletion and tumor removal	Melanoma	Autoimmune vitiligo establishes durable anti‐melanoma immunity in the skin by inducing the generation and maintenance of melanoma‐specific CD103^+^ T_RM_ cells through adoptive transfer	[228]
	Inhibition of fatty acid oxidation therapy (Etomoxir) ^+^Anti‐PD‐L1 mAb	Gastric cancer	PD‐L1 blockade alters lipid metabolism by decreasing FABP4/5 expression in gastric adenocarcinoma cells and increasing it in CD8^+^ T_RM_ cells, enhancing survival and antitumor immunity through improved lipid uptake	[295]
	The small molecule inhibitor iBFAR2 targeting BFAR	Bladder, breast, prostate cancer, melanoma	BFAR inhibits cytokine‐induced JAK2 signaling by promoting the deubiquitination of JAK2, thereby limiting T_RM_ cell reprogramming mediated by STAT1	[344]
iPSC‐derived therapy	HLA editing	Cervical cancer	By expressing HLA‐A24 and HLA‐E to evade immune rejection, and being rich in tissue‐resident memory T cells, thus enhancing cytotoxicity against cervical cancer	[352]

Abbreviations: TLR9, Toll‐like receptor 9; HPV, human papillomavirus; FABP4/5, fatty acid‐binding protein 4/5; BFAR, bifunctional apoptosis regulator.

iPSC‐T_RM_ cells combine stem cell scalability with the tissue‐persistent properties of T_RM_ cells. Furukawa et al. demonstrate that human papillomavirus‐specific cytotoxic T lymphocytes (HPV‐rejTs)—iPSC‐derived, T_RM_‐enriched cytotoxic T cells—exhibit potent anti‐cervical cancer activity [348, 349]. To overcome allogeneic rejection, CRISPR‐Cas9 scarless editing was used to generate HLA‐A24 and HLA‐E dual‐integrated HPV‐rejTs, minimizing immune rejection while retaining cytotoxicity [350, 351]. High T_RM_ content enhances antitumor function via TGF‐βR‐driven CD103 upregulation and immunological synapse gene activation [352, 353, 354, 355], supporting an “off‐the‐shelf” T‐cell therapy for cervical cancer. iPSC‐derived T_RM_ cell therapies represent a cutting‐edge and promising approach in cancer immunotherapy, offering unlimited “off‐the‐shelf” T cells capable of regenerating antitumor immunity.

## 7 Conclusion and Future Prospects

The significance of human CD8^+^ T_RM_ cells across a spectrum of diseases is increasingly acknowledged, as cutting‐edge high‐throughput sequencing and spatiotemporal omics technologies thoroughly dissect their developmental dynamics and heterogeneity [94]. CD8^+^ T_RM_ cells have been demonstrated to originate from antigen‐experienced T cell populations primed during early childhood development. These cells undergo progressive, site‐specific maturation throughout infancy and late childhood, gradually acquiring unique tissue‐resident characteristics at phenotypic, functional, and transcriptional levels [356]. CD8^+^ T_RM_ cells exhibit marked functional and phenotypic heterogeneity across and within tissues, presenting both challenges for precise characterization and opportunities for refined therapeutic targeting. Deciphering this diversity is crucial for elucidating tumor immune surveillance mechanisms, T‐cell trafficking dynamics, and predictors of immunotherapy response. CD8^+^ T_RM_ cells display substantial functional and phenotypic heterogeneity across and within tissues, posing challenges for mechanistic studies and therapeutic development while offering opportunities for precision immunotherapy. The heterogeneity of T_RM_ cells is not randomly generated but is spatiotemporally imprinted by the regionalized structure of the tissue microenvironment [94, 120, 357]. Resolving this complexity is critical to advance our understanding of tumor immune surveillance mechanisms, tissue‐specific T‐cell trafficking, and predictors of treatment response.

CD8^+^ T cells co‐expressing residency and exhaustion molecules (e.g., PD‐1 and CD39) in chronic diseases, including chronic infection, autoimmunity, and cancers, often correlate with clinical outcomes [222, 358, 359]. These cells are often classified as T_RM_ cells based on expression of tissue‐residency molecules such as CD69 and CD103 [24, 360, 361]. However, the relationship between these cells and conventional CD8^+^ T_RM_ cells or exhausted CD8^+^ T_EX_ cells is unclear. To address the long‐standing uncertainty, a recent study defines a novel tissue‐resident T_EX_ cell (TR‐T_EX_) subset that is ontogenetically and functionally distinct from conventional T_RM_ cells [362]. T_RM_ and TR‐T_EX_ are distinct CD8^+^ T cell subsets arising from divergent differentiation trajectories. TR‐T_EX_ cells originate from the T_EX_ progenitor cell (T_EX‐PROG_) during chronic infection. While sharing the core tissue‐residency program (Runx3, Itgae/CD103) with canonical T_RM_ cells, they exhibit heightened activity of exhaustion‐associated regulators (Tox, Eomes, Pdcd1/PD‐1, Entpd1/CD39) [362, 363, 364]. This distinct phenotype positions TR‐T_EX_ cells—rather than conventional T_RM_ cells—as preferential targets for immune checkpoint blockade (ICB). Clinical evidence demonstrates that the TR‐T_EX_ transcriptional signature (but not the T_RM_ signature) correlates with: (1) improved treatment response in metastatic melanoma patients receiving anti‐PD‐1/CTLA‐4 therapy, and (2) enhanced survival following anti‐PD‐L1 intervention [362, 365]. Together, these findings challenge the traditional understanding and classification of PD‐1^+^ T_RM_ cells, highlighting the need for further in‐depth research to elucidate their precise biological characteristics and regulatory mechanisms [366].

The regulatory role of CD4^+^ T_RM_ cells is increasingly recognized in a wide array of diseases. After acute viral infection, memory CD4^+^ T cells predominantly survey nonlymphoid tissues via tissue residence, but this process is not as stringent as that seen in CD8^+^ T cells [367]. In contrast with CD8^+^ T_RM_ cells, CD4^+^ T_RM_ cells are retained in deeper tissue layers, and CD103 is not part of the core T_RM_ signature in these cells, thus implying distinct residency programs [367, 368]. CD4^+^ T helper (T_H_) T_RM_ cells are important in assisting local development of protective memory B and CD8^+^ T cell responses [369]. Moreover, CD4^+^ T_RM_ cells exert their instrumental role in immune control of *M. tuberculosis* infection in the human lung through the production of IL‐17 [370]. CD4^+^ T_RM_ cells also form in response to parasitic infection. Upon stimulation with *Leishmania*, they produce IFN‐γ. During a secondary challenge, they serve as sentinels that rapidly recruit circulating memory cells, leading to enhanced protection against reinfection [371, 372]. Collectively, CD4^+^ T_RM_ cells reside at mucosal and barrier surfaces, including the respiratory and gastrointestinal tracts. They react swiftly upon rechallenge and coordinate local immune responses to defend against pathogens.

A few studies have investigated the kinetics of CD8^+^ T_RM_ cells formation and decay, as well as the T_RM_ density essential for protection [6, 373]. T_RM_ decay kinetics are highly organ‐specific, with stable persistence in the salivary gland, biphasic decay in the small intestine (fast phase, *t*_1/2_ = 41 days and slow phase, *t*_1/2_ = 633 days), and continuous attrition in the uterus (*t*_1/2_ = 82 days) [374]. Lung CD8^+^ T_RM_ cells undergo rapid apoptosis‐dependent decay, declining 500‐fold between days 50 and 200 after influenza infection, and heterosubtypic protection is completely lost by day 125 [375]. Despite these promising advances, many critical questions remain to be answered on the way to translating T_RM_‐targeted interventions into clinical practice. For example, can we establish stable, functional T_RM_ cells in target human tissues? How to balance the efficacy and safety of T_RM_‐based therapies in clinical settings? How to develop selective modulation strategies to enhance protective T_RM_ cells without activating pathogenic subsets? These questions represent a key direction for future research and will require extensive efforts.

In conclusion, we comprehensively summarize the characteristics and functions of CD8^+^ T_RM_ cells, reviewing their regulatory functions in health and disease immunity. Notably, therapeutic strategies targeting CD8^+^ T_RM_ cell regulation—supported by robust preclinical models and validated in clinical trials—have shown promising therapeutic efficacy in specific disease contexts. Critically, advances in CD8^+^ T_RM_ cell biology have provided fundamental insights into memory T cell diversification, reinforcing their tissue‐residency programs and functional plasticity while unlocking novel translational avenues for precision immunotherapy development. We hope this review will provide fundamental and comprehensive references for subsequent studies in the field.

## Author Contributions

J.Y., Y.A., and X.Z. designed the review; J.Y., Y.A., X.Z., T.X., S.L., M.X., Y.L. (Yunxia Lin), H.M., Y.L. (Yue‐yang Liu), Y.W., L.Y., H.M., Y.L. (Yong‐da Liu), and C.Z. searched for literature; J.Y., Y.A., T.X., X.Z., S.L., and M.X. wrote and revised the manuscript. JY and XZ provided funding support. All authors have read and approved the article.

## Funding

This work was supported by the National Natural Science Foundation of China (Grant Nos. 92374119, 82422059, 32470973, 92059111, 82421005, and 81972738), Noncommunicable Chronic Diseases‐National Science and Technology Major Project (2023ZD0500102), and the University Experimental Technology Team Construction Plan of Shanghai.

## Ethics Statement

The authors have nothing to report.

## Conflicts of Interest

The authors declare no conflicts of interest

## Data Availability

Not applicable.
